# The Potential Effect of Metformin on Cancer: An Umbrella Review

**DOI:** 10.3389/fendo.2019.00617

**Published:** 2019-09-18

**Authors:** Hong Yu, Xi Zhong, Peng Gao, Jinxin Shi, Zhonghua Wu, Zhexu Guo, Zhenning Wang, Yongxi Song

**Affiliations:** Department of Surgical Oncology and General Surgery, The First Hospital of China Medical University, Shenyang, China

**Keywords:** metformin, cancer, umbrella review, drug re-purposing, decision-making

## Abstract

**Background:** Metformin has been reported to possess anti-cancer properties in addition to glucose-lowering activity and numerous systematic reviews and meta-analyses have studied the association between metformin use and cancer incidence or survival outcomes. We performed an umbrella review to assess the robustness of these associations to facilitate proper interpretation of these results to inform clinical and policy decisions.

**Methods:** We searched PubMed and Embase systematic reviews and meta-analyses investigating the effect of metformin use on cancer incidence or survival outcomes published from inception to September 2, 2018. We estimated the summary effect size, the 95% CI, and the 95% prediction interval, heterogeneity, evidence of small-study effects, and evidence of excess significance bias.

**Results:** We included 21 systematic reviews and meta-analyses covering 11 major anatomical sites and 33 associations. There was strong evidence for the association between metformin use and decreased pancreatic cancer incidence. The association between metformin use and improved colorectal cancer overall survival (OS) was supported by highly suggestive evidence. Seven associations (all cancer incidence, all cancer OS, breast cancer OS, colorectal cancer incidence, liver cancer incidence, lung cancer OS, and pancreatic cancer OS) presented only suggestive evidence. The remaining 24 associations were supported by weak or not-suggestive evidence.

**Conclusions:** Associations between metformin use and pancreatic cancer incidence or colorectal cancer OS are supported by strong or highly suggestive evidence, respectively. However, these results should be interpreted with caution due to the poor methodological quality of the systematic reviews and meta-analyses.

## Introduction

Cancer is one of the largest problems in the world at present, ranking second among all factors causing death in the United States every year (1). Current treatment methods for cancer include surgical resection, radiotherapy, and chemotherapy. Due to rapid tumor cell growth, easy metastasis, chemoradiotherapy resistance, and other biological characteristics, it is difficult for conventional treatments to completely remove tumor cells, contributing to poor prognosis. Drug re-purposing has been used to ensure the safety of drugs and avoid long cycles of drug development and screening. Today, some well-established drugs such as aspirin (2) and digoxin (3) have been found to have anti-tumor effects and have been applied in new fields.

Metformin is a semi-synthetic oral hypoglycemic agent which mainly reduces blood glucose by activating the adenosine monophosphate activated protein kinase (AMPK) signaling pathway, inhibiting hepatic glucose output, improving peripheral tissue sensitivity to glucose, and increasing glucose uptake (4). The definite curative effect, good safety, and low cost have allowed metformin to be recommended as the first-line oral treatment for type 2 diabetes mellitus (T2DM) and the most commonly prescribed drug in T2DM patients. In 1998, British scientists found that metformin had a protective effect on the cardiovascular system (5), inspiring researchers to work on re-purposing metformin. Subsequent studies reported that metformin can be used for adjuvant treatment of tuberculosis (6) and for routine treatment of polycystic ovary syndrome (7).

In recent years, epidemiological data have shown that diabetes increases the risk of breast cancer, colorectal cancer, pancreatic cancer, endometrial cancer, and other malignant tumors. In 2005, Scottish researchers found that diabetic patients taking metformin had a lower risk of cancer, indicating that metformin may possess anti-tumor abilities (8). According to another study, metformin likely has an inhibitory effect on tumor progression in patients with T2DM, which can reduce the risk of tumor and tumor-related mortality of patients, improving their survival rate (9). An increasing number of observational studies and randomized controlled trials (RCT) have studied the association between metformin use and cancer incidence or survival outcomes (10), and a mass of systematic reviews and meta-analyses have been conducted to evaluate the relationship between metformin and cancer (11). The observed associations between may exaggerate the effects of metformin on cancer, as substantial heterogeneity and potential biases reside in the included systematic reviews. We performed an umbrella review to provide a comprehensive review of claimed associations between metformin use and cancer risk or survival outcomes for different cancers and critically assess the robustness of these associations to facilitate proper interpretation of these results to inform clinical and policy decisions.

## Methods

### Protocol and Study Design

The protocol for conducting this umbrella review of systematic reviews and meta-analyses exploring the effect of metformin use on cancer risk or survival outcomes was developed accordingly:

### Search Strategy and Eligibility Criteria

Two researchers (HY and XZ) independently searched PubMed and Embase systematic reviews and meta-analyses investigating the effect of metformin use on cancer incidence or survival outcomes published from inception to September 2, 2018. The search strategy used the following terms: metformin AND (cancer OR tumor OR neoplasm OR malignan^*^) AND (systematic OR meta-analysis). Two authors (HY, XZ) independently reviewed the titles and abstracts, carefully read the full text of potential eligible studies, and completed the study selection.

Included eligible systematic reviews and meta-analyses addressed associations between metformin use and cancer incidence and/or survival outcomes, including overall survival (OS), recurrence-free survival (RFS), cancer-specific survival (CSS), and progression-free survival (PFS). Articles were also included if studies assessed different groups of people with the same cancer or focused on subtypes of a particular cancer. When two or more meta-analyses were found on the same association, only the one containing the most primary studies was included to avoid duplication.

### Data Extraction

Two investigators (HY, XZ) independently extracted data from eligible articles, retrieving first author name; publication year; cancer type; number of included studies; number of cases and population size; relative risk estimates including risk ratio (RR), odds ratio (OR), and hazard ratio (HR); and the corresponding 95% confidence interval (CI) from the eligible systematic reviews and meta-analyses. Divergences were resolved through discussion. First author, number of cases and population size, relative risk estimates (RR, OR, and HR), and corresponding 95% CI were extracted from each individual study in included systematic reviews or meta-analyses for further analysis.

### Quality Assessment

Each included systematic review and meta-analysis was independently assessed by two authors (HY, XZ) using the Assessing the Methodological Quality of Systematic Reviews version 2.0 (AMSTAR 2.0) tool (12). AMSTAR 2.0 measures 16 items, provides a comprehensive rationale for item selection, identifies critical domains, and rates the validity of the results of the review as high, moderate, low, or critically low instead of creating an overall score. These features make AMSTAR 2.0 a major upgrade to AMSTAR. Discrepancies were resolved by consensus.

### Statistical Analysis

#### Assessment of Summary Effect and Heterogeneity

For each meta-analysis on the association between metformin use and cancer risk or survival outcomes, the summary effect was synthesized and its 95% CI was calculated using random-effect models. Inter-study heterogeneity was evaluated with Cochran's *Q*-test and the *I*^2^ statistic (13). Statistical inconsistencies could demonstrate either genuine inter-study heterogeneity or underlying bias. The 95% CI of *I*^2^ was calculated to assess the uncertainty around heterogeneity estimates (14).

#### Estimation of Prediction Intervals

Ninety five percentage prediction intervals (PI) for the summary random effect estimates were calculated to further interpret inter-study heterogeneity and represent the prediction of the effect in an individual-study setting (15).

#### Assessment of Small-Study Effects

Small study effects can indicate publication bias, genuine heterogeneity, or chance. The Egger's regression asymmetry test was used to detect small study effect biases. Small study effect bias was considered to exist when the Egger's test *P* < 0.10 (16).

#### Evidence of Excess Significance Bias

The excess significance test evaluated whether the actual observed number (O) of positive studies (*P* < 0.05) was different from the expected number of studies (E) with statistically significant results (17). E was the sum of the statistical power estimates for each component study in each meta-analysis and was calculated with an algorithm using a non-central t distribution. In cases in which *O* > *E* AND *P* < 0.10, the excess significance test was considered positive.

#### 10% Credibility Ceiling

Credibility ceiling analyses were performed to account for the innate methodological limitations of observational studies (18). The level was set at 10% to re-estimate the inter-study heterogeneity and summary relative risk between studies.

### Grading the Existing Evidence

Nominally statistically significant (*P* < 0.05) associations from meta-analyses exploring the effect of metformin use on cancer risk or survival outcomes were classified into four levels—strong [*P* < 10^−6^, >1,000 cases, *P* <0.05 of the largest component study in the meta-analysis, no large heterogeneity (*I*^2^ < 50%), no evidence of small-study effects (*P* > 0.1 for Egger's test), the 95% PI excludes the null value (1), no excess significance bias (*P* > 0.1), and survives the 10% credibility ceiling test (*P* > 0.05)], highly suggestive (*P* < 10^−6^, >1,000 cases, *P* < 0.05 of the largest component study in the meta-analysis), suggestive (*P* < 10^−3^, >1,000 cases), and weak (*P* < 0.05) (19, 20).

All statistical analyses were performed using STATA version 12.0.

## Results

### Characteristics of the Included Systematic Reviews and Meta-Analyses

**Figure 1:**
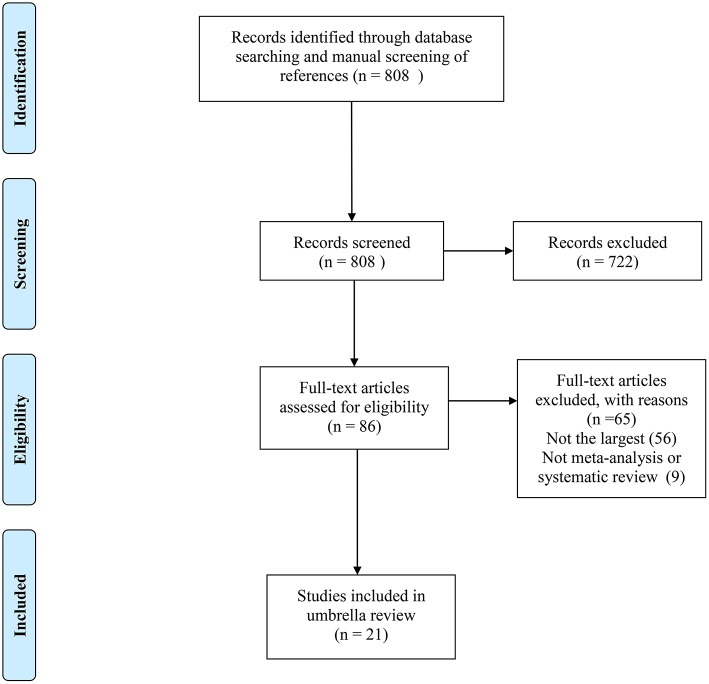
The flow diagram of study selection.

**Figure 2:**
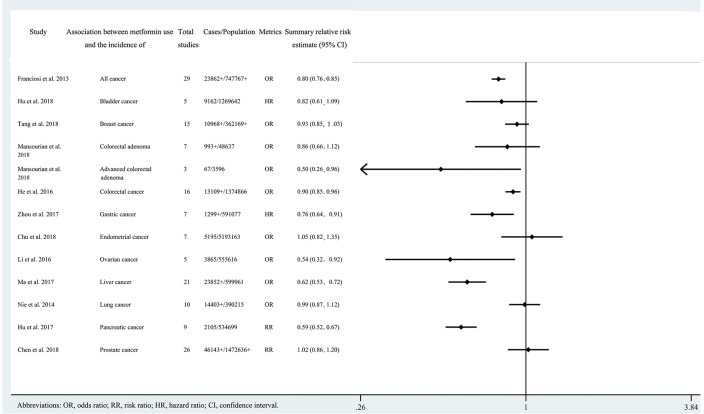
Characteristics of the 13 associations on cancer incidence in the included systematic reviews and meta-analyses.

**Figure 3:**
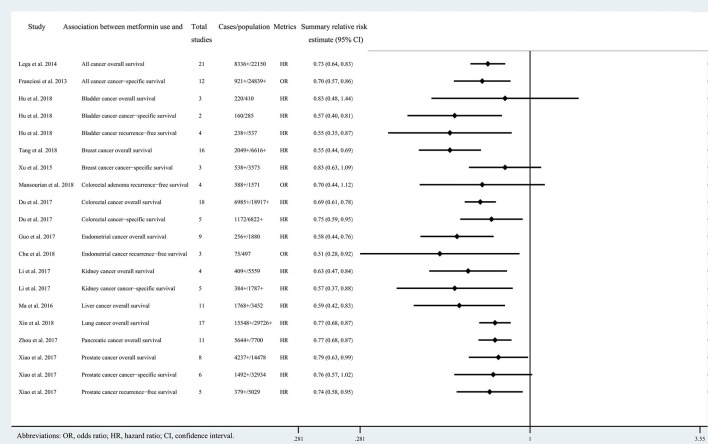
Characteristics of the 20 associations on cancer prognosis in the included systematic reviews and meta-analyses.

Of the 808 records obtained from the literature search through PubMed and Embase, we ultimately included 21 systematic reviews covering 33 associations (11, 21–40). The search flowchart and reasons for excluding 787 are shown in Figure 1. The 21 studies included 11 major anatomical sites (bladder, breast, colorectum, gastric, endometrium, ovarian, kidney, liver, lung, pancreas and prostate), 33 different associations between metformin use and cancer risk or survival outcomes, 327 primary studies, more than 206,000 cases and more than 13 million subjects. Of note, all subjects were diagnosed with T2DM at baseline. The characteristics of the 33 associations are shown in Figures 2, 3, and the full database of the 327 primary studies is available in Supplementary Table 4.

### Quality Assessment Using AMSTAR 2.0

We used the 16-item AMSTAR 2.0 to assess the methodological quality of the 21 eligible systematic reviews, and all qualities were considered critically low. All included studies had more than one critical flaw [usually in items 2 (18/21, 85.7%), 7 (21/21, 100%), and 13 (21/21, 100%)] and several non-critical flaws [usually in items 3 (18/21, 85.7%), 10 (21/21, 100%), and 12 (19/21, 90.5%)]. It should be noted that reviews with more than one critical flaw, regardless of non-critical weaknesses, should be interpreted with caution for a credible summary of the existing evidence. The detailed results, scoring criteria, and rating criteria are shown in Supplementary Table 1.

### Summary Effect Size

Twenty three of the 33 associations in the included meta-analyses were statistically significant with a threshold of *P* < 0.05, with the remaining 10 associations presenting *P* > 0.05. Of the statistically significant associations, five reached *P* < 10^−6^: associations between metformin use and pancreatic cancer incidence, colorectal cancer OS, all cancer incidence, breast cancer OS, or liver cancer incidence (Tables 1, 2; Supplementary Tables 2, 3). Associations between metformin use and all cancer OS, colorectal cancer incidence, lung cancer OS, pancreatic cancer OS, all cancer CSS, or endometrial cancer OS reached a moderate statistical significance (*P* < 10^−3^). The remaining 12 associations including bladder cancer CSS, bladder cancer RFS, advanced colorectal adenoma incidence, colorectal cancer CSS, gastric cancer incidence, endometrial cancer RFS, ovarian cancer incidence, kidney cancer OS, kidney cancer CSS, liver cancer OS, prostate cancer OS and prostate cancer RFS reached *P* < 0.05. The combined results demonstrate that metformin can decrease cancer risk or increase survival outcome among all associations with strongly statistically significant summary random effect estimates.

**Table 1:** Evidence-rating results based on the results of statistical analyses of the 13 associations on cancer incidence.

**Study**	**Association between metformin use and the incidence of**	Summary relative risk estimate (random-effect *P*)^*^	**Cases** **> 1,000**	**Largest study** **relative risk estimate *P* < 0.05**	***I*^**2**^** **< 50%**	**Small** **study effects**	**95% prediction interval exclude the null value**	**Excess significance**	**Ten percentage credibility** **ceiling survival**
Associations supported by strong evidence (1)									
Hu et al. (38)	Pancreatic cancer	+++	+	+	+	–	+	–	+
Associations supported by suggestive evidence (3)									
Franciosi et al. (21)	All cancer	+++	+	–	–	+	–	+	–
He et al. (27)	Colorectal cancer	++	+	+	+	–	–	–	–
Ma et al. (34)	Liver cancer	+++	+	–	–	+	–	+	+
Associations supported by weak evidence (3)									
Mansourian et al. (26)	Advanced colorectal adenoma	+	–	+	–	–	–	–	–
Zhou et al. (29)	Gastric cancer	+	+	+	–	+	–	+	–
Li et al. (32)	Ovarian cancer	+	+	+	–	–	–	–	–
Associations supported by not suggestive evidence (6)									
Hu et al. (23)	Bladder cancer	–	+	+	–	+	–	–	–
Tang et al. (24)	Breast cancer	–	+	+	+	–	–	–	–
Mansourian et al. (26)	Colorectal adenoma	–	–	+	–	+	–	–	–
Chu et al. (30)	Endometrial cancer	–	+	+	–	–	–	–	–
Nie et al. (36)	Lung cancer	–	+	+	–	–	–	–	–
Chen et al. (11)	Prostate cancer	–	+	+	–	+	–	–	–

* *P-value calculated using random–effect model: +++, P < 10^−6^; ++, P < 10^−3^; +, P < 0.05; –, P > 0.05. For other items, + = yes, – = no*.

**Table 2:** Evidence-rating results based on the results of statistical analyses of the 20 associations on cancer prognosis.

**Study**	**Association between metformin use and**	Summary relative risk estimate (random-effect *P*)^*^	**Cases** **>1000**	**Largest study** **relative risk estimate *P* < 0.05**	***I*^**2**^** **< 50%**	**Small** **study effects**	**95% prediction interval exclude the null value**	**Excess significance**	**10% credibility** **ceiling survival**
Associations supported by highly suggestive evidence (1)									
Du et al. (28)	Colorectal cancer overall survival	+++	+	+	–	+	–	–	+
Associations supported by suggestive evidence (4)									
Lega et al. (22)	All cancer overall survival	++	+	–	–	+	–	+	+
Tang et al. (24)	Breast cancer overall survival	+++	+	–	–	+	–	+	+
Xin et al. (37)	Lung cancer overall survival	++	+	+	–	–	–	–	+
Zhou et al. (29)	Pancreatic cancer overall survival	++	+	–	–	–	–	–	+
Associations supported by weak evidence (11)									
Franciosi et al. (21)	All cancer cancer-specific survival	++	–	+	+	–	–	–	+
Hu et al. (23)	Bladder cancer cancer-specific survival	+	–	+	–	#	#	+	–
Hu et al. (23)	Bladder cancer recurrence-free survival	+	–	–	–	–	–	+	–
Du et al. (28)	Colorectal cancer cancer-specific survival	+	+	+	–	–	–	–	–
Guo et al. (31)	Endometrial cancer overall survival	++	–	+	+	–	–	–	–
Chu et al. (30)	Endometrial cancer recurrence-free survival	+	–	–	+	–	–	–	+
Li et al. (33)	Kidney cancer overall survival	+	–	–	+	–	–	–	+
Li et al. (33)	Kidney cancer cancer-specific survival	+	–	–	+	–	–	–	+
Ma et al. (35)	Liver cancer overall survival	+	+	–	–	–	–	+	+
Xiao et al. (40)	Prostate cancer overall survival	+	+	+	–	–	–	–	–
Xiao et al. (40)	Prostate cancer recurrence-free survival	+	–	–	+	–	–	–	–
Associations supported by not suggestive evidence (4)									
Hu et al. (23)	Bladder cancer overall survival	–	–	–	–	–	–	+	–
Xu et al. (25)	Breast cancer cancer-specific survival	–	–	–	+	–	–	–	–
Mansourian et al. (26)	Colorectal adenoma recurrence-free survival	–	–	–	–	–	–	–	–
Xiao et al. (40)	Prostate cancer cancer-specific survival	–	+	+	–	–	–	–	–

* *P-value calculated using random-effect model: +++, P < 10^−6^; ++, P < 10^−3^; +, P < 0.05; –, P > 0.05. For other items, + = yes, – = no*.

^#^*Hu et al. (23) included only two primary studies, thus Egger's test and prediction interval are not available*.

### Heterogeneity

There were 10 associations with moderate to high heterogeneity (*I*^2^ = 50–75%) and 12 associations with high heterogeneity (*I*^2^ > 75%). When we calculated the 95% PI to further assess inter-study heterogeneity, we found only one association with the null value excluded (pancreatic cancer incidence) (Tables 1, 2; Supplementary Tables 2, 3).

### Small-Study Effects

Of the 33 associations between metformin use and cancer incidence or survival outcomes, small study effects were detected in nine (colorectal cancer OS, all cancer incidence, all cancer OS, breast cancer OS, liver cancer incidence, gastric cancer incidence, colorectal adenoma incidence, bladder cancer incidence and prostate cancer incidence) according to the Egger's test (*P* < 0.1) as shown in Tables 1, 2; Supplementary Tables 2, 3.

### Excess Significance

Nine associations (all cancer incidence, all cancer OS, breast cancer OS, liver cancer incidence, bladder cancer CSS, bladder cancer RFS, gastric cancer incidence, liver cancer OS, and bladder cancer OS) had evidence of excess significance bias (*P* < 0.1 AND *O* > *E*) (Tables 1, 2; Supplementary Tables 2, 3).

### 10% Credibility Ceiling

Twelve associations (pancreatic cancer incidence, colorectal cancer OS, all cancer OS, breast cancer OS, liver cancer incidence, lung cancer OS, pancreatic cancer OS, all cancer CSS, endometrial cancer RFS, kidney cancer OS, kidney cancer CSS, and liver cancer OS) remained statistically significant with credibility ceilings set at 10%.

### Robustness of Evidence

We found that 24 of 33 associations between metformin use and cancer risk or survival outcomes were supported by weak or not-suggestive evidence (Tables 1, 2; Supplementary Tables 2, 3). There was strong evidence for the association between metformin use and pancreatic cancer incidence. The association between metformin use and colorectal cancer OS was supported by highly suggestive evidence. The remaining 7 associations (all cancer incidence, all cancer OS, breast cancer OS, colorectal cancer incidence, liver cancer incidence, lung cancer OS, and pancreatic cancer OS) presented only suggestive evidence.

## Discussion

### Main Findings and Interpretation in Light of Existing Evidence

In this umbrella review of systematic reviews and meta-analyses evaluating the current evidence for associations between metformin use and cancer risk or survival outcomes, we summarized 21 studies covering 11 major anatomical sites, 327 primary studies, more than 206,000 cases, and over 13 million subjects. According to statistical data analyses, the association between metformin and pancreatic cancer incidence was supported by strong evidence, suggesting that metformin may be associated with decreased risk of pancreatic cancer. We additionally found that the association between metformin and colorectal cancer OS had highly suggestive evidence, indicating that patients with colorectal cancer using metformin likely have a better OS than those not using metformin.

Pancreatic cancer ranks fourth for cancer-related deaths worldwide (41). However, just 10–20% of the patients are eligible for surgical treatment because of the late diagnosis (42). Several studies showed significantly reduced risk of pancreatic cancer in T2DM patients treated with metformin compared with those without (43, 44). It is reported that metformin inhibits proliferation of pancreatic cancer cells through the AMPK/mammalian target of rapamycin (mTOR) axis and by down-regulating activity of the insulin/insulin-like growth factor signaling pathway (45, 46). In our study, evidence for associations between metformin and pancreatic cancer was strong for incidence and suggestive for OS. Therefore, policy makers should cautiously consider metformin for routine use to protect against pancreatic cancer or as an option in treating pancreatic cancer patients, especially those with T2DM.

Colorectal cancer ranks as the third most common malignant tumor in the world. About 600 thousand people die of colorectal cancer every year worldwide (47). It has been reported that metformin intake is associated with reduced risk of colorectal cancer and improved survival in colorectal cancer patients (48, 49). These results are consistent with the findings in our study as we found highly suggestive evidence for colorectal cancer OS and suggestive evidence for colorectal cancer risk. Our findings may shed light on the treatment or prevention of colorectal cancer with metformin.

With the development of medical science and the continuous discussion and practice of drugs, drug repurposing refers to the drugs that have been put on the market for a long time and have been known by the majority of medical groups, seek new medical treatments from among existing medications rather than through the development of *de novo* medicines (50). Drug repurposing is based on previous research and development. Detailed formulation, mechanism and safety information of drugs are known, which means that compared with brand-new drugs, old drugs with new uses have obvious advantages of low research cost, low risk and high success rate, and can be put into clinical trials more quickly. Drug repurposing is a kind of innovation in the deep exploration of pharmacological mechanism in clinical practice. As a drug development strategy, it has received more and more attention, and a large number of new drugs for indications have been born. For example, dapoxetine for premature ejaculation is based on drug side effects; thalidomide for the treatment of multiple myeloma is based on the existing mechanism.

Metformin is a safe and effective biguanide hypoglycemic agent, which mainly activates AMPK signaling pathway, decreases hepatic glucose output, promotes the uptake of glucose in peripheral tissues, increases insulin sensitivity, inhibits intestinal cells from absorbing glucose, promotes the secretion of GLP-1 (51) and affect gut microbiota (52). Recent studies have found that metformin has the effects of weight loss, anti-aging and anti-cardiovascular disease. With the increasing awareness of metformin on cancer, numerous studies have shown that metformin can be used to reduce the incidence of cancer and improve the prognosis of cancer patients. Diabetes patients taking metformin reduced their risk of cancer by 30 to 50 percent, especially the risk of pancreatic, hepatocellular, and colon cancers (53).

There have been substantial reports on the mechanism of metformin's anti-tumor effect. Metformin activates AMPK and induces G1 phase arrest of the cell cycle by inhibiting cyclin D1 expression (54). Activation of AMPK can increase the expression of p53 gene and play an anti-tumor role. Low concentration of metformin can induce p53-dependent cell senescence of liver cancer cells by activating AMPK (55). Metformin can also inhibit tumor growth through the mTOR signaling in AMPK-dependent and -independent pathway (56). Treatment with metformin lowers serum levels of insulin and insulin-like growth factor-1 (IGF-1), which are both potential growth factors capable of stimulating cell survival and mitogenesis (57, 58). Furthermore, a recent study has reported the effect of metformin on androgen-induced IGF-I receptor (IGF-IR) upregulation resulting in the reduction of IGF1-mediated biological effects in prostate cancers cells (59). Normal cells generate adenosine-triphosphate (ATP) mainly through mitochondrial oxidative phosphorylation, while most tumor cells prefer anaerobic glycolysis as an energy generation approach, which is the Warburg effect (60). AMPK activation inhibits fatty acid synthetase and acetyl-CoA carboxylase, leading to reduced fatty acid production in tumor cells, which inhibits tumor cells proliferation. In the study of the tricarboxylic acid cycle, molecular oxygen is reduced to deuterium depleted water, which affects gluconeogenesis as well as fatty acid oxidation, by the terminal complex of mitochondrial electron transport chain (61). Deuterium depleted water is proposed to delay tumor progression using natural ketogenic diets and low deuterium drinking water (62). This action is shared by other biguanides (such as phenformin), statins, and gleevec based on their structural similarities (63). These findings may open up new oncology directions toward metabolically driven submolecular targets to prevent and treat cancers.

Previous studies have shown that high glucose can provide the optimal growth environment for tumors. On the other hand, the hypoglycemic effect of metformin may mask the regulatory effect of metformin on related pathways in tumor therapy. Therefore, it is worth considering whether metformin is effective in patients with pre-diabetes, normal blood glucose or even hypoglycemia. Elgendy et al. (64), found that intermittent fasting in combination with metformin can inhibit the glycolysis and oxidative phosphorylation of tumor cells, namely the drug showing the best antitumor effect with low blood glucose level. At the meanwhile, the strongest anti-tumor effect of metformin at hypoglycemia is independent of AMPK activity. Zhuang et al. (65) also found that low glucose can enhance the toxicity of metformin in breast and ovarian cancer cells and reduce the intracellular ATP level. However, considering the literature body of current evidence, the application of metformin to patients with normal blood glucose remains to be discussed. The effects of metformin on patients' blood glucose and the side effects of metformin are all worthy of clinicians' caution.

### Strengths and Limitation

We performed this detailed umbrella review to evaluate the evidence of associations between metformin and cancer risk or survival outcomes and applied statistical analyses to assess robustness and validity. In addition, we used a comprehensive and systematic criterion to grade evidence levels to rate the strength of these systematic reviews and meta-analyses.

Our review inevitably has limitations and drawbacks. First, we fully trust the accuracy of the data provided in the included meta-analyses. As such, problems within the published data may impact the evidence-rating results despite our statistical analyses. Second, meta-analyses that include <10 studies did not allow for statistical tests to identify small study effects and excess significance. Third, the methodological quality of all included systematic reviews and meta-analyses were considered critically low, and future studies that meet the stringent criteria of AMSTAR 2.0 should be conducted to further confirm the findings in our study.

## Conclusion

In conclusion, associations between metformin use and pancreatic cancer incidence or colorectal cancer OS are supported by strong or highly suggestive evidence, respectively. However, these results should be interpreted with caution and no firm conclusion can be drawn due to the poor methodological quality of the systematic reviews and meta-analyses.

## Data Availability

All datasets generated for this study are included in the Supplementary Table 4.

## Author Contributions

YS, ZWa, and XZ conceived and designed the study. HY and XZ performed the literature search, acquired, and collated the data, which were analyzed by XZ, PG, JS, ZWu, and ZG. All authors drafted, critically revised the manuscript for important intellectual content, and gave final approval of the version to be published and contributed to the manuscript. YS and ZWa are guarantors. YS and ZWa attest that all listed authors meet authorship criteria and that no others meeting the criteria have been omitted.

### Conflict of Interest Statement

The authors declare that the research was conducted in the absence of any commercial or financial relationships that could be construed as a potential conflict of interest.
