# Supplementary material for: The Potential Effect of Metformin on Cancer: An Umbrella Review
Source: Front Endocrinol (Lausanne). 2019 Sep 18;10:617. doi: 10.3389/fendo.2019.00617 (PMC6760464; doi:10.3389/fendo.2019.00617)
Supplement: Supplementary file 2 [file Table_2.docx]

Supplementary Table 2. Results of statistical analyses for the evidence rating of the 13 associations on cancer incidence

| Study | Association between metformin use and the incidence of | Summary relative risk (random effect) | | Cases | Largest study | I^2^  (95% CI) | *P* value for Egger’s test | 95% prediction interval | Excess significance test | | 10% credibility ceiling  (*P* value) |
| --- | --- | --- | --- | --- | --- | --- | --- | --- | --- | --- | --- |
|  |  | Estimate | *P*  value | *N* | Relative risk estimate (95% CI) |  |  |  | *O/E* | *P*  value |  |
| Franciosi et al, 2013^a^ | All cancer | 0.80 (0.76-0.85) | 2.80*10^-13^ | 23862^d^ | 1.00 (0.99-1.00) | 96%  (96-97%) | 0.008 | (0.64-1.01) | 11/4.3 | 4.64*10^-4^ | 0.13 |
| Hu et al, 2018^c^ | Bladder cancer | 0.82  (0.61–1.09) | 0.17 | 9162 | 0.60 (0.57-0.64) | 85%  (62-92%) | 0.072 | (0.29-2.28) | 1/4.22 | 7.23*10^-5^ | 0.26 |
| Tang et al, 2018^a^ | Breast cancer | 0.93 (0.85-1.03) | 0.16 | 10968^d^ | 0.95 (0.91-0.99) | 35%  (0-64%) | 0.28 | (0.75-1.17) | 4/7.40 | 0.08 | 0.06 |
| Mansourian et al, 2018^a^ | Colorectal adenoma | 0.86  (0.66-1.12) | 0.27 | 993^d^ | 1.17  (1.08-1.27) | 72%  (20-85%) | 0.047 | (0.39-1.90) | 3/1.95 | 0.38 | 0.55 |
| Mansourian et al, 2018^a^ | Advanced colorectal adenoma | 0.50  (0.26-0.96) | 0.04 | 67 | 0.49 (0.39-0.62) | 63%  (0-87%) | 0.80 | (0.00-591) | 2/1.25 | 0.38 | 0.16 |
| He et al, 2016^a^ | Colorectal cancer | 0.90  (0.85-0.96) | 7.93*  10^-4^ | 13109^d^ | 0.91 (0.88-0.94) | 47%  (0-69%) | 0.40 | (0.77-1.06) | 5/5.32 | 0.87 | 0.05 |
| Zhou et al, 2017^c^ | Gastric cancer | 0.76  (0.64–0.91) | 2.02*10^-3^ | 1299^d^ | 0.99 (0.98-0.99) | 92%  (86-95%) | 0.049 | (0.46-1.27) | 5/1.30 | 3.23*10^-4^ | 0.17 |
| Chu et al, 2018^a^ | Endometrial cancer | 1.05 (0.82-1.35) | 0.70 | 5195 | 0.68 (0.61-0.74) | 91%  (84-94%) | 0.84 | (0.44-2.52) | 4/6.25 | 5.97*10^-3^ | 0.58 |
| Li et al, 2016^a^ | Ovarian cancer | 0.54 (0.32-0.92) | 0.02 | 3865 | 0.34 (0.31-0.37) | 85%  (60-92%) | 0.23 | (0.09-3.11) | 1/4.9 | 1.26*10^-35^ | 0.028 |
| Ma et al, 2017^a^ | Liver cancer | 0.62  (0.53-0.72) | 2.11*10^-9^ | 23852^d^ | 0.99 (0.99-1.00) | 89%  (85-92%) | <0.001 | (0.38-1.00) | 10/2.05 | 5.06*10^-9^ | 0.003 |
| Nie et al, 2014^a^ | Lung cancer | 0.99 (0.87-1.12) | 0.86 | 14403^d^ | 0.87 (0.84-0.91) | 80%  (62%-88%) | 0.22 | (0.66-1.49) | 4/4.74 | 0.64 | 0.98 |
| Hu et al, 2017^b^ | Pancreatic cancer | 0.59  (0.52–0.67) | 4.56*10^-15^ | 2105 | 0.64 (0.56-0.74) | 30%  (0-67%) | 0.81 | (0.44-0.79) | 6/6.22 | 0.87 | 0.008 |
| Chen et al, 2018^b^ | Prostate cancer | 1.02  (0.86–1.20) | 0.83 | 46143^d^ | 0.48 (0.46-0.50) | 97%  (97%-98%) | 0.02 | (0.43-2.41) | 10/24.95 | 3.63*10^-50^ | 0.58 |

a Reported odds ratio (OR);

b Reported risk ratio (RR);

c Reported hazard ratio (HR);

d contain missing values.

*N*= number; *O*= the number of observed statistically significant studies; *E*= the number of expected statistically significant studies.
