# Supplementary material for: The Potential Effect of Metformin on Cancer: An Umbrella Review
Source: Front Endocrinol (Lausanne). 2019 Sep 18;10:617. doi: 10.3389/fendo.2019.00617 (PMC6760464; doi:10.3389/fendo.2019.00617)
Supplement: Supplementary file 3 [file Table_3.docx]

Supplementary Table 3. Results of statistical analyses for the evidence rating of the 20 associations on cancer prognosis

| Study | Association between metformin use and | Summary relative risk (random effect) | | Cases | Largest study | I^2^  (95% CI) | *P* value for Egger’s test | 95% prediction interval | Excess significance test | | 10% credibility ceiling  (*P* value) |
| --- | --- | --- | --- | --- | --- | --- | --- | --- | --- | --- | --- |
|  |  | Estimate | *P*  value | *N* | Relative risk estimate (95% CI) |  |  |  | *O/E* | *P*  value |  |
| Lega et al, 2014^c^ | All cancer overall survival | 0.73 (0.64-0.83) | 1.29*  10^-6^ | 8336^d^ | 0.97 (0.92-1.02) | 82%  (74-87%) | 0.017 | (0.45-1.18) | 15/5 | 3*10^-7^ | 0.02 |
| Franciosi et al, 2013^a^ | All cancer cancer-specific survival | 0.70 (0.57-0.87) | 9.96*10^-4^ | 921^d^ | 0.80  (0.65-0.98) | 27%  (0-63%) | 0.69 | (0.45-1.10) | 4/4.92 | 0.59 | 0.02 |
| Hu et al, 2018^c^ | Bladder cancer overall survival | 0.83 (0.48-1.44) | 0.51 | 220 | 1.13  (0.70-1.80) | 64%  (0-88%) | 0.98 | (0-361.94) | 1/0.20 | 0.06 | 0.99 |
| Hu et al, 2018^c^ | Bladder cancer cancer-specific survival | 0.57  (0.40-0.81) | 1.51*10^-3^ | 160 | 0.57 (0.36-0.91) | 0%  （-） | - | - | 2/0.70 | 0.05 | 0.07 |
| Hu et al, 2018^c^ | Bladder cancer recurrence-free survival | 0.55 (0.35-0.88) | 0.01 | 238^d^ | 1.00 (0.62-1.60) | 64%  (0-86%) | 0.19 | (0.08-3.78) | 3/1.15 | 0.04 | 0.19 |
| Tang et al, 2018^c^ | Breast cancer overall survival | 0.55 (0.44-0.70) | 5.46*  10^-7^ | 2049^d^ | 0.97 (0.92-1.02) | 81%  (69-87%) | <0.001 | (0.24-1.29) | 11/7.49 | 0.08 | 0.001 |
| Xu et al, 2015^c^ | Breast cancer cancer-specific survival | 0.83 (0.63-1.08) | 0.16 | 538^d^ | 0.91 (0.81-1.03) | 46%  (0-84%) | 0.40 | (0.05-12.85) | 1/1.25 | 0.77 | 0.12 |
| Mansourian et al, 2018^a^ | Colorectal adenoma recurrence-free survival | 0.70  (0.44–1.12) | 0.14 | 588^d^ | 0.94 (0.79-1.11) | 55%  (0-83%) | 0.15 | (0.12-4.13) | 1/1.19 | 0.84 | 0.26 |
| Du et al, 2017^c^ | Colorectal cancer overall survival | 0.69 (0.61-0.77) | 3.09*  10^-10^ | 6985^d^ | 0.79 (0.73-0.86) | 74%  (55-82%) | 0.037 | (0.44-1.06) | 13/10.30 | 0.20 | 3.4*  10^-4^ |
| Du et al, 2017^c^ | Colorectal cancer-specific survival | 0.75  (0.59–0.94) | 0.01 | 1172 | 0.66 (0.54-0.81) | 53%  (0-81%) | 0.97 | (0.37-1.50) | 2/3.40 | 0.18 | 0.18 |
| Guo et al, 2017^c^ | Endometrial cancer overall survival | 0.58  (0.44–0.76) | 8.96*10^-5^ | 256^d^ | 0.49 (0.34-0.71) | 27%  (0-66%) | 0.15 | (0.32-1.06) | 4/6.14 | 0.13 | 0.058 |
| Chu et al, 2018^a^ | Endometrial cancer recurrence-free survival | 0.51 (0.28-0.92) | 0.03 | 75 | 0.52 (0.20-1.35) | 0%  (0-72%) | 0.54 | (0.01-25.05) | 0/1.08 | 0.19 | 0.04 |
| Li et al, 2017^c^ | Kidney cancer overall survival | 0.63 (0.47-0.84) | 1.86*10^-3^ | 409^d^ | 0.77 (0.57-1.05) | 41%  (0-79%) | 0.76 | (0.22-1.77) | 2/3.39 | 0.05 | 0.049 |
| Li et al, 2017^c^ | Kidney cancer cancer-specific survival | 0.57  (0.37-0.88) | 0.01 | 384^d^ | 0.78 (0.50-1.23) | 29%  (0-73%) | 0.30 | (0.19-1.72) | 1/1.76 | 0.48 | 0.04 |
| Ma et al, 2016^c^ | Liver cancer overall survival | 0.59  (0.42-0.83) | 2.47*10^-3^ | 1768^d^ | 1.00 (0.80-1.30) | 83%  (69-89%) | 0.68 | (0.18-1.89) | 8/4.35 | 0.02 | 0.046 |
| Xin et al, 2018^c^ | Lung cancer overall survival | 0.77  (0.69–0.86) | 6.22*10^-6^ | 15548^d^ | 0.86 (0.81-0.91) | 70%  (45-80%) | 0.15 | (0.53-1.11) | 10/13.4 | 0.04 | 4.8*  10^-4^ |
| Zhou et al, 2017^c^ | Pancreatic cancer overall survival | 0.77  (0.68–0.87) | 3.52*10^-5^ | 5644^d^ | 0.92 (0.79-1.08) | 53%  (0-74%) | 0.13 | (0.54-1.09) | 5/4.51 | 0.76 | 0.008 |
| Xiao et al, 2017^c^ | Prostate cancer overall survival | 0.79  (0.63–0.98) | 0.036 | 4237^d^ | 0.76 (0.70-0.82) | 79%  (54-88%) | 0.98 | (0.40-1.54) | 5/4.96 | 0.98 | 0.19 |
| Xiao et al, 2017^c^ | Prostate cancer cancer-specific survival | 0.76 (0.57-1.02) | 0.07 | 1492^d^ | 0.76 (0.64-0.89) | 65%  (0-84%) | 0.32 | (0.35-1.68) | 3/4.11 | 0.33 | 0.28 |
| Xiao et al, 2017^c^ | Prostate cancer recurrence-free survival | 0.74 (0.58-0.95) | 0.02 | 379^d^ | 0.91 (0.67-1.24) | 29%  (0-74%) | 0.24 | (0.39-1.41) | 1/2.25 | 0.26 | 0.11 |

a Reported odds ratio (OR);

c Reported hazard ratio (HR);

d contain missing values.

- Hu et al, 2018 included only two primary studies, thus Egger’s test and prediction interval are not available.

*N*= number; *O*= the number of observed statistically significant studies; *E*= the number of expected statistically significant studies.
